# Frequency of SARS-CoV-2 variants identified by real-time PCR in the AUNA healthcare network, Peru

**DOI:** 10.3389/fpubh.2023.1244662

**Published:** 2024-02-12

**Authors:** Tamin Ortiz-Gómez, Andrea C. Gomez, Brigitte Chuima, Alejandra Zevallos, Karen Ocampo, Diana Torres, Joseph A. Pinto

**Affiliations:** ^1^Laboratorios AUNA, Área de Biología Molecular, Lima, Peru; ^2^Centro de Investigación Básica y Translacional, AUNA IDEAS, Lima, Peru; ^3^Escuela Profesional de Medicina Humana, Universidad Privada San Juan Bautista, Lima, Peru; ^4^Escuela Profesional de Medicina Humana, Universidad Privada Norbert Wiener, Lima, Peru

**Keywords:** SARS-CoV-2 variants, surveillance & forecast system, epidemiological monitoring, RT-PCR method, COVID-19

## Abstract

**Introduction:**

In Peru, on 11 February 2023, the Ministry of Health registered 4 million patients infected with COVID-19 and around 219,260 deaths. In 2020, the SARS-CoV-2 virus was acquiring mutations that impacted the properties of transmissibility, infectivity, and immune evasion, leading to new lineages. In the present study, the frequency of COVID-19 variants was determined during 2021 and 2022 in patients treated in the AUNA healthcare network.

**Methods:**

The methodology used to detect mutations and identify variants was the Allplex™ SARS-CoV-2 Variants Assay I, II, and VII kit RT-PCR. The frequency of variants was presented by epidemiological weeks.

**Results:**

In total, 544 positive samples were evaluated, where the Delta, Omicron, and Gamma variants were identified. The Delta variant was found in 242 (44.5%) patients between epidemiological weeks 39 and 52 in 2021. In the case of Gamma, it was observed in 8 (1.5%) patients at weeks 39, 41, 43, 45, and 46 of 2021. The Omicron variant was the most frequent with 289 (53.1%) patients during weeks 49 to 52 of 2021 and 1 to 22 of 2022. During weeks 1 through 22 of 2022, it was possible to discriminate between BA. 1 (*n* = 32) and BA.2 (*n* = 82).

**Conclusion:**

The rapid identification of COVID-19 variants through the RT-PCR methodology contributes to timely epidemiological surveillance, as well as appropriate patient management.

## Introduction

SARS-CoV-2 is an emerging virus that made its first appearance in the Chinese province of Wuhan in November 2019 (1). It is alarming that the World Health Organization (WHO) declared it a global pandemic (2). In Peru, on 5 March 2020, the first case of COVID-19 was reported. Today, more than 4 million cases of COVID-19 have been confirmed in Peru and approximately 219,587 deaths were registered (3).

SARS-CoV-2 has constantly changed, this caused the virus to acquire mutations in the spike protein gene (S-gene) giving new lineages of origin of SARS-CoV-2. These mutations have an impact on the properties of the virus, such as transmissibility, immune response, infectivity, and disease severity (4). WHO and the Center for Disease Control and Prevention (CDC) of the United States classified these variants as Variant of concern (VOC): Alpha, Beta, Gamma, Delta, and the currently circulating variant of concern Omicron and Variants of interest (VOI): Iota, Eta, Kappa, Epsilon, Lambda, and Zeta (5).

The SARS-CoV-2 lineages have been determined by whole genome sequencing (WGS). This test has been used since the start of the pandemic to gather essential information for public health. WGS requires high-complexity laboratories, highly qualified staff, expensive resources, and a long time for analysis (6). The implementation of real-time PCR in laboratories became a great alternative for monitoring circulating variants because it can be used as a rapid screening for epidemiological surveillance (7, 8). Additionally, real-time PCR has advantages such as the rapid process time, the affordable price, and the easy implementation in a molecular biology laboratory. In the present study, we describe the frequency of SARS-CoV-2 variants in the Auna healthcare network from Peru determined by real-time identification of the S-gene mutation.

## Materials and methods

### Samples and selection criteria

Genetic materials were obtained from nasopharyngeal swabs from September to December (39 to 52 epidemiological weeks) in 2021 and from January to May (1 to 22 epidemiological weeks) in 2022, which were previously tested for SARS-CoV-2 by reverse transcription PCR (RT-PCR) (9, 10). We stored remnant RNA at −80°C from positive samples with a cycle threshold (CT) value less than 30. All procedures were performed in the Molecular Biology area of the Auna Laboratory.

### Real-time PCR for variant determination

The identification of the variants was performed with real-time PCR using a commercial kit. The Allplex™ SARS-CoV-2 Variants I Assay detects and identifies the HV69/70 deletion, E484K and N501Y mutations of the S gene and the Allplex™ SARS-CoV-2 Variants II Assay detects the mutations: L452R, W152C, K417T, and K417N. Together, this allows the identification of the VOC: Alpha, Beta, Gamma, Delta, and Omicron (11, 12). The differentiation between the lineages of Omicron BA.1 and BA.2 was determined using the Novaplex™ SARS-CoV-2 Variants VII Assay. This assay detects mutations: N501Y, E484A and HV69/70 deletion (13).

### Whole genome sequencing

The national programme for genomic surveillance of the SARS-CoV-2 virus in Peru is directed by the National Institute of Health (NIH) with the objective of developing adequate genomic surveillance of the circulation of variants of SARS-CoV-2. As an authorised laboratory, we participated in the analytical surveillance programme, which allowed us to know the exact lineage of some samples with the methodology of whole genome sequencing. A total of 49 samples were derived from the INS Genomic Laboratory of the INS; these results were compared with those obtained using the real-time PCR methodology for VOC identification.

### Statistical analysis

The data analysis of the multiplex real-time PCR was developed using the automated software Seegene viewer. This allows identification and differentiation for both the Ct value and multiple targets in a single channel. The information for this epidemiological study was collected from the laboratory software Enterprise. The data was analysed in R studio and presented in graphs and tables.

## Results

**Figure 1:**
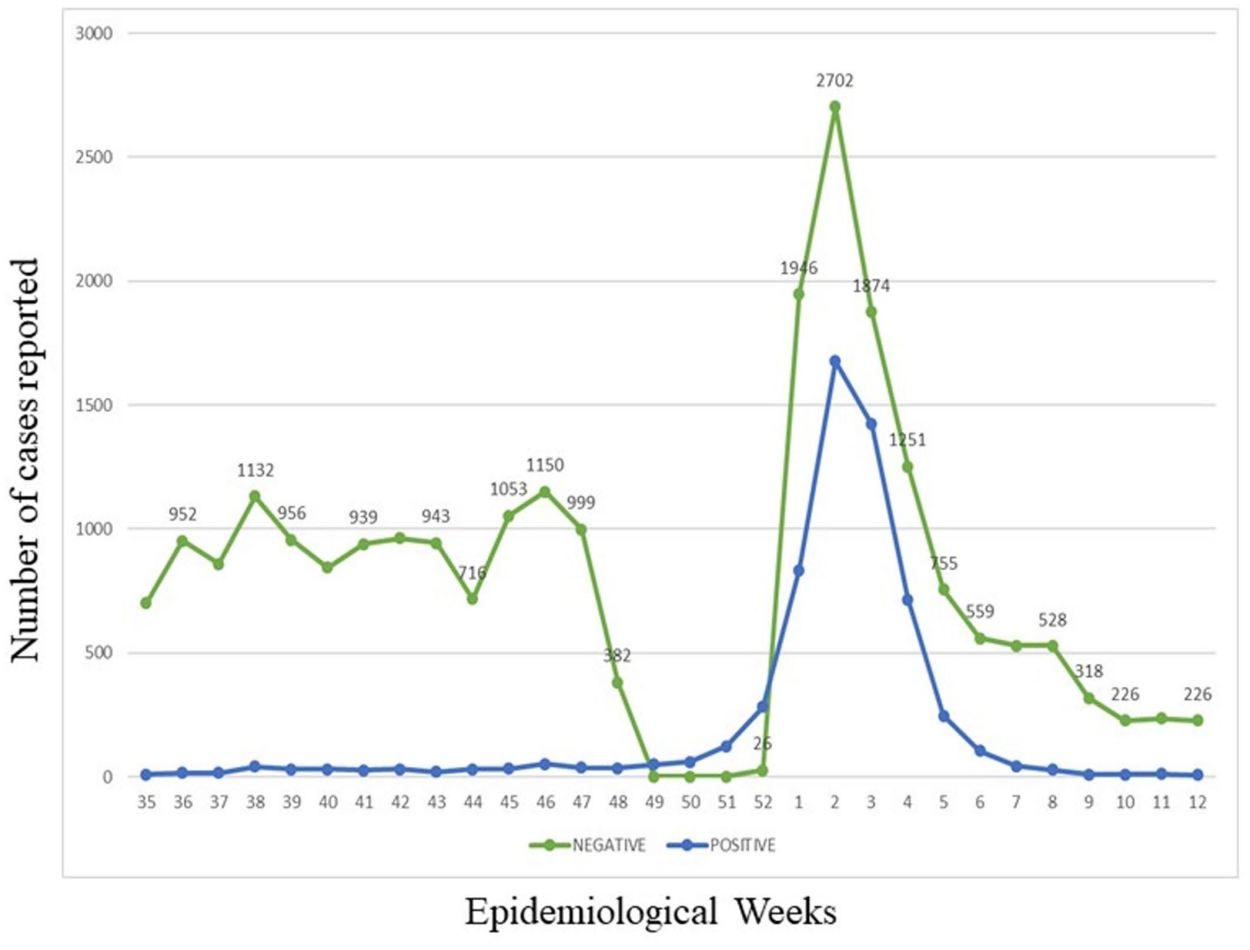
Evolution over the time of all samples processed from September to December 2021 and January to May 2022. The green line reports the COMD-19 negative cases. The blue line reports the COMD-19 positive cases.

The sampling was carried out in the Auna clinic network from September to December 2021 and from January to May 2022. During this time, around 31,375 samples were analysed, reaching 50% positivity during the third wave in Peru between December 2021 and February 2022 (Figure 1).

**Figure 2:**
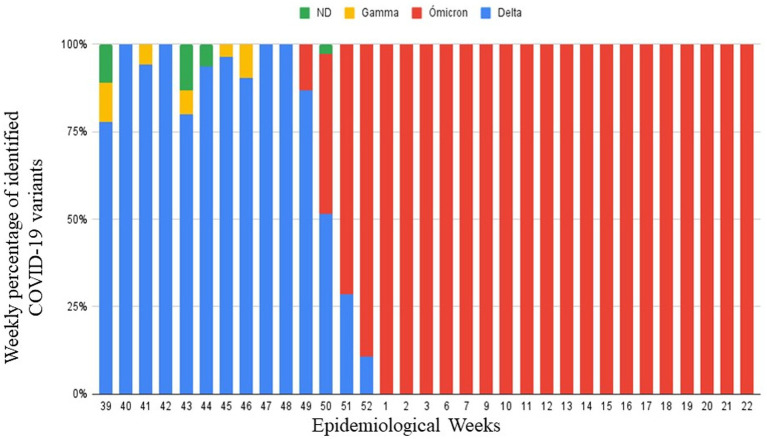
Identified variants by real-time PCR during epidemiological weeks 39–52 in 2021 and epidemiological weeks 1–22 in 2022.

A total of 544 samples with CT values less than 30 were selected, of which 432 ARN samples were tested with Allplex™ MC SARS-CoV-2 Variants I and II and 112 ARN samples were tested with Novaplex™ MC SARS-CoV-2 Variants VII (Tables 1, 2). As shown in Figure 2, the Delta variant was found during 39 to 52 weeks in 2021 with 242 (44.5%) positive samples. The Omicron variant was found in both years, 49 to 22 epidemiological weeks in 2021 and 1 to 22 epidemiological weeks in 2022. Overall, there were 289 samples with Omicron (53.1%). Gamma was the variant with the lowest frequency with 8 (1.5%) positive samples. Our data showed that in five samples, we were unable to identify the variant.

**Table 1:** Analysis of SARS-CoV-2 variants in samples obtained from AUNA patients.

Sample code	Peru-NIH results		I and II variants				AUNA results
	Lineage	Variant	N501Y	HV69/70 del	L452R	K417N	
17,306	B.1.617.2	Delta			+		Delta
17,274	BA.1	Omicron	+	+		+	Omicron
17,345	AY.99	Delta			+		Delta
17,162	AY.9.2	Delta			+		Delta
17,140	BA.1	Omicron	+	+		+	Omicron
16,971	AY.39.2	Delta			+		Delta
16,786	AY.39.2	Delta			+		Delta
16,594	AY.39.2	Delta			+		Delta
16,464	B.1.617.2	Delta			+		Delta
16,448	AY.119.1	Delta			+		Delta
17,456	BA.1	Omicron	+	+		+	Omicron
17,458	C.37	Lambda					Unidentified
17,468	BA.1	Omicron	+	+		+	Omicron
17,690	BA.1	Omicron	+	+		+	Omicron
17,693	BA.1	Omicron	+	+		+	Omicron
17,755	AY.25	Delta			+		Delta
17,777	AY.3	Delta			+		Delta
17,790	BA.1	Omicron	+	+		+	Omicron
17,827	BA.1	Omicron	+	+		+	Omicron
17,835	BA.1	Omicron	+	+		+	Omicron
18,379	BA.1	Omicron	+	+		+	Omicron
18,380	AY.122	Delta			+		Delta
18,694	BA.1	Omicron	+	+		+	Omicron
18,698	AY.119.1	Delta			+		Delta
18,699	BA.1	Omicron	+	+		+	Omicron
18,812	BA.1	Omicron	+	+		+	Omicron
18,861	BA.1	Omicron	+	+		+	Omicron
18,862	BA.1	Omicron	+	+		+	Omicron
18,863	BA.1	Omicron	+	+		+	Omicron
18,864	AY.122	Delta			+		Delta
18,865	BA.1	Omicron	+	+		+	Omicron
18,791	BA.1	Omicron	+	+		+	Omicron
18,792	BA.1	Omicron	+	+		+	Omicron
18,620	BA.1	Omicron	+	+		+	Omicron
18,677	AY.119.1	Delta			+		Delta
18,948	BA.1	Omicron	+	+		+	Omicron
34,631	BA.1	Omicron	+	+		+	Omicron
34,789	BA.1	Omicron	+	+		+	Omicron
34,832	BA.1	Omicron	+	+		+	Omicron
34,940	BA.1	Omicron	+	+		+	Omicron
35,011	BA.1.1	Omicron	+	+		+	Omicron

Comparison between the sequencing conducted at Peru-NIH with the typing conducted in Auna with Allplex™ SARS-CoV-2 Variants I Assay and Allplex™ SARS-CoV-2 Variants II Assay (I and II variants).

**Table 2:** Analysis of SARS-CoV-2 variants in samples obtained from AUNA patients.

Sample code	Peru-NIH results		Novaplex™ SARS-CoV-2 Variants VII				AUNA results
	Lineage	Variant	HV69/70 del	E484A	510Y		
37,771	BA.2	Omicron		+	+		Omicron BA.2
37,839	BA.2.9	Omicron		+	+		Omicron BA.2
37,608	BA.2	Omicron		+	+		Omicron BA.2
37,688	BA.2	Omicron		+	+		Omicron BA.2
37,758	BA.2	Omicron		+	+		Omicron BA.2
37,817	BA.2.12.1	Omicron		+	+		Omicron BA.2
37,831	BA.2	Omicron		+	+		Omicron BA.2
38,006	BA.2	Omicron		+	+		Omicron BA.2

Comparison between the sequencing conducted at Peru-NIH with the typing conducted in Auna with NovaplexTM MC SARS-CoV-2 Variants VII kit.

**Figure 3:**
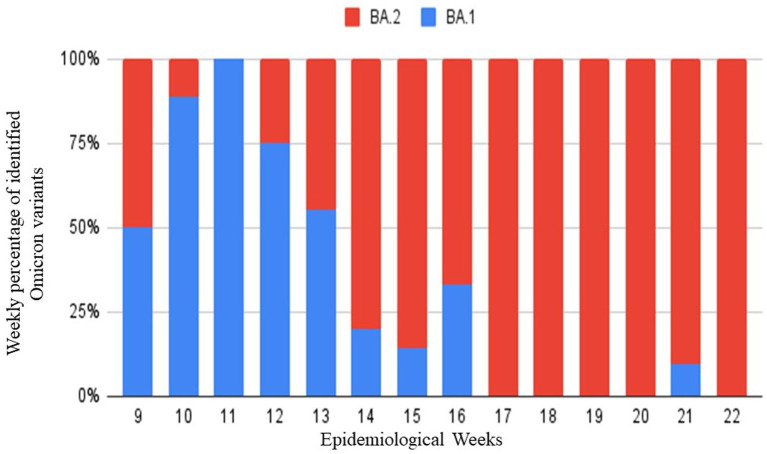
Identified Omicron variants during epidemiological weeks 9 to 22 of 2022 by real-time PCR with Novap1ex™ SARS-CoV-2 Variants VII Assay. In red BA.2 variant and in Blue BA.I variant.

The Omicron variant was identified between BA.1 and BA.2 at weeks 9 to 22 of 2022. BA.2 was the most frequent, with 80 (71.4%) samples identified. Unlike BA.1, with 32 (28.6%) positive samples. As shown in Figure 3, in the last weeks, BA.2 was the most frequent.

As part of the COVID-19 surveillance in Peru, the National Institute of Health (NIH) requested samples from positive patients from laboratories that performed COVID-19 tests. Tables 1, 2 show the samples sent from 2021 to 2022 to the NIH. Only one sample could not be identified by real-time PCR used in the Auna laboratory. However, the NIH identified it as the Lambda variant by WGS. Furthermore, in the Supplementary Table S1, it can be seen that in all other samples Auna achieved the same diagnosis as the NIH.

## Discussion

During the pandemic, COVID-19 developed different variants that caused worldwide concern. In Peru, the third wave of COVID-19 was declared in the 49 and 50 epidemiological weeks in 2021, increasing to more than 50% of positive cases (14, 15). In our study, Figure 1, we found an increasing number of positive cases of COVID-19 in December 2021 and January 2022, which were the months with the highest positive cases.

In Figure 2, we show the circulating variants in our area during the last trimester of 2021 and the first semester of 2022. The results obtained during 39 to 49 weeks were consistent with the end of the second wave in Peru, which had a predominance of delta variants. Also, it can be seen that from week 50 of 2021, Omicron began to appear in patients. However, from week 52 of 2021 to week 22 of 2022, Omicron shows a rapid progression that becomes predominant in all the cases. In Figure 3, from weeks 9 to 22 of 2022, Omicron began to be identified between its appearances BA.1 and BA.2 appearances, where it is evident that BA.2 had the highest prevalence. These changes are based on the local epidemiology of Peru and the recovered collected data for the INS that make Omicron responsible for the third wave (16, 17).

According to the NGS results shown in Table 1, using both Seegene Variants I and II assays allowed to identify VOC in all cases. However, these assays do not identify VOI as in the case of lambda (12, 17). Our study shows five cases with a nonidentified result; those cases are presumptive other variants different from VOC. This amount represents the 0.9% of samples analysed. In addition to that, with the emergence of Omicron subvariants, the Variants I and II assays do not discriminate between Omicron BA.1 and BA.2. The transition from BA.1 to BA.2 could be detected using the Variant VII Assay during weeks 9 to 22 of 2022.

Different assays were evaluated to identify VOC and VIC during the COVID-19 pandemic. The Allplex Seegene Variants kits showed concordance rates with WGS between 96.4 and 98.10% (18). Additionally, the Thermo Fisher TaqPath COVID-19 PCR assay achieved a 98.2% success rate in variant identification (19). The TaqPath methodology provides the advantage of identifying multiple mutations based on the current context, unrestricted to a specific kit. The high mutation rate of SARS-CoV-2 have led to the implementation of a surveillance programme in Peru to identify variants responsible for each wave. Gamma, Lambda and Delta variants principally detected during the second wave, and Omicron variant responsible for the third wave. Similar scenarios have been observed worldwide, but the timing and number of waves vary across regions (20–22). Peru has experienced diverse circulating variants, including native variants like Lambda, considered endemic to our territory (23, 24).

On the other hand, as the population immunity to SARS-CoV-2 increases, a variety of mutations have been identified that enhance replication fitness and have given rise to the VOCs. First, D614G spike protein mutation is in the C-terminal region of S1 domain related to S2. This mutation provokes a rapidly spread, higher transmissibility and more pathogenicity (25, 26). N501Y mutation enhances ACE2 proximity, it is on the RBD increasing the affinity to host cell receptor and increasing the transmission (Alpha variant) (27, 28). E484K mutation is involved in the initiation of the viral entry process and occurs at critical sites in the receptor binding motif of RBD. This mutation can escape from neutralization that affects the antibody-based measuring the monoclonal antibodies and vaccines (Beta variant) (28). On the other hand, NTD mutations has a deletion at 69–70 position that has shown a higher viral replication (29, 30). At the position 141–146 and 242–244, this deletion causes the neutralization of NTD antibodies. The reduction of the susceptibility to NTD neutralizing antibodies is produced by L18F, D253Y and other NTD mutilations, such the case of Gamma variant (29, 31). Non-spike mutations have been observed to enhance the transitivity of the virus host cells (32).

Aditionally, the multiple variants of SARS-CoV-2 that have surfaced recently have created uncertainty regarding the disease and its effects on vaccination programs. According to clinical trial evidence, vaccines currently available against the alpha variant (B.1.1.7) are highly effective (33–37). In the case of beta variants (B.1.351), some vaccines have shown reduced efficacy and effectiveness against symptomatic disease (38–41). Similarly, the effectiveness and efficacy of some vaccines have been reduced against delta variants (B.1.617.2) (42, 43). However, they have remained effective against severe disease and hospitalization (44–48). According to data on the effectiveness of vector-based vaccines against omicron variant (B.1.1.529), primary vaccine regimes fail to provide adequate protection (49). Likewise, primary mRNA vaccination regimens provide insufficient protection (50). However, it is important to highlight that vaccines remain effective against severe disease and hospitalization caused by the omicron variant, with initial data indicating 90% effectiveness against hospitalization from the United Kingdom (51, 52). Finally, it is noteworthy to mention that in the case of vaccine variants that do not have immune escape capability, a high-efficacy vaccine may only need a booster dose, whereas a lower-efficacy vaccine may need to be adapted. Thus, it is possible that all vaccines will require adaptation for variants with a significant immune escape ability, such as the Omicron variant. Equally, it is noteworthy to mention that artificial intelligence and big data have great potential for COVID-19 and other emergencies surveillance, and their roles are expected to expand over the next few years. Using AI and Big Data, public health interventions can be planned and organized based on the spread of the virus, their effectiveness monitored, old compounds can be repurposed and new drugs discovered, vaccine candidates can be identified, and communities and territories can be better prepared for the outbreak. Currently, a few studies have shown that AI-assisted methods distinguish accurately between different viral strains, aiding targeted interventions and surveillance (53–57). However, For AI-assisted methods to be effective in real world situations, more work should be done on strengthening the representativeness of data, improving predictive capabilities, and integrating AI-assisted methods into existing public health settings.

This study presents some limitations. First, the small number of samples collected during the selection of the third wave. Second, the high impact in the positive cases and the storage capacity of the laboratory complicated the recollection of ARN. However, the results obtained are based on local epidemiology during this time. Third, the kits Allplex™ SARS-CoV-2 Variants I and II assay were limited to the detection of Gamma, Beta, Delta, and Omicron that were considered VOCs (11, 12, 18). Since the SARS-CoV-2 virus is constantly mutational, these essays could quickly become out of date. For this reason, RT-PCR methods must be in permanent actualization (20). Finally, RT-PCR kits focus on a select group of mutations that identify a variant using an interpretative algorithm. Additional information like lineage, genomic diversity, and genotyping must be obtained by WGS (21).

In the present study, data from the official epidemiological surveillance issued by the NIH are correlated with national data obtained during the epidemiological weeks monitored. The data obtained during the study helped to observe the evolution of the mutations present in the second and third waves in our country. On the other hand, the kits used for RT-PCR identification have 98% reproducible genomic sequencing. Furthermore, the kits allowed us to identify variants of COVID-19 at a much lower cost and with much less equipment than the NGS method.

In conclusion, our data show the landscape of variant transitions in a private clinic where the omicron variant eventually replaced all circulating SARS-CoV-2 variants. In our experience, although with limited data, the determination of SARS-CoV-2 variants through RT-PCR assays was reliable and useful in routine.

## Data availability statement

The datasets presented in this study can be found in the Figshare repository https://figshare.com/s/9e8295978679d692a711.

## Ethics statement

The studies involving humans were approved by the IRB of the Universidad Privada San Juan Bautista (Approval: 0600-2023-CIEI-UPSJB), which waived the requirement of written informed consent for participation from the participants or the participants’ legal guardians/next of kin because a database was used with patients’ personal information codified. The studies were conducted in accordance with the local legislation and institutional requirements.

## Author contributions

TO-G: design of the study, laboratory analysis, data creation, writing draft, and review and editing. AG: formal analysis, data curation, writing draft, and review and editing. BC: design of the study, laboratory analysis, and review and editing. AZ: ethics committee approval procedures, writing draft, and review and editing. KO: laboratory analysis, data creation, and review and editing. DT: laboratory analysis, data creation, and review and editing. JP: design of the study, formal analysis, writing draft, and review and editing. All authors contributed to the article and approved the submitted version.

## References

[1] Huang C Wang Y Li X Ren L Zhao J Hu Y. Clinical features of patients infected with 2019 novel coronavirus in Wuhan, China. Lancet. (2020) 395:497–506. doi: 10.1016/S0140-6736(20)30183-5, PMID: 31986264 PMC7159299

[2] World Health Organization. WHO director-General's opening remarks at the media briefing on COVID-19. (2020). Available at: https://www.who.int/director-general/speeches/detail/who-director-general-s-opening-remarks-at-the-media-briefing-on-covid-19---11-march-2020 (Accessed May 9, 2023).

[3] Ministry of Health, Peru. Sala COVID-19. (2023). Available at: https://www.dge.gob.pe/covid19.html (Accessed May 9, 2023).

[4] Volz E Hill V McCrone JT Price A Jorgensen D O'Toole Á. Evaluating the effects of SARS-CoV-2 spike mutation D614G on transmissibility and pathogenicity. Cells. (2021) 184:64–75.e11. doi: 10.1016/j.cell.2020.11.020, PMID: 33275900 PMC7674007

[5] Centers for Disease Control and Prevention. Clasificaciones y definiciones de las variantes del SARS-CoV-2. (2023). Available at: https://espanol.cdc.gov/coronavirus/2019-ncov/variants/variant-classifications.html (Accessed May 9, 2023).

[6] Greninger AL Dien Bard J Colgrove RC Graf EH Hanson KE Hayden MK. Clinical and infection prevention applications of severe acute respiratory syndrome coronavirus 2 genotyping: an Infectious Diseases Society of America/American Society for Microbiology consensus review document. J Clin Microbiol. (2022) 60:e0165921. doi: 10.1128/JCM.01659-21, PMID: 34731022 PMC8769737

[7] Neopane P Nypaver J Shrestha R Beqaj SS. SARS-CoV-2 variants detection using TaqMan SARS-CoV-2 mutation panel molecular genotyping assays. Infect Drug Resist. (2021) 14:4471–9. doi: 10.2147/IDR.S335583, PMID: 34737587 PMC8558424

[8] Borillo GA Kagan RM Marlowe EM. Rapid and accurate identification of SARS-CoV-2 variants using real time PCR assays. Front Cell Infect Microbiol. (2022) 12:894613. doi: 10.3389/fcimb.2022.894613, PMID: 35619652 PMC9127862

[9] Sule WF Oluwayelu DO. Real-time RT-PCR for COVID-19 diagnosis: challenges and prospects. Pan Afr Med J. (2020) 35:121. doi: 10.11604/pamj.supp.2020.35.24258, PMID: 33282076 PMC7687508

[10] Rahbari R Moradi N Abdi M. rRT-PCR for SARS-CoV-2: analytical considerations. Clin Chim Acta. (2021) 516:1–7. doi: 10.1016/j.cca.2021.01.011, PMID: 33485902 PMC7826022

[11] Fu JYL Chong YM Sam IC Chan YF. SARS-CoV-2 multiplex RT-PCR to detect variants of concern (VOCs) in Malaysia, between January to may 2021. J Virol Methods. (2022) 301:114462. doi: 10.1016/j.jviromet.2022.114462, PMID: 35026305 PMC8744358

[12] Umunnakwe CN Makatini ZN Maphanga M Mdunyelwa A Mlambo KM Manyaka P. Evaluation of a commercial SARS-CoV-2 multiplex PCR genotyping assay for variant identification in resource-scarce settings. PLoS One. (2022) 17:e0269071. doi: 10.1371/journal.pone.0269071, PMID: 35749403 PMC9231807

[13] Ministry of Health, Peru. Minsa confirma tercera ola ante incremento de casos de contagio por la COVID-19. (2022). Available at: https://www.gob.pe/institucion/minsa/noticias/574040-minsa-confirma-tercera-ola-ante-incremento-de-casos-de-contagio-por-la-covid-19 (Accessed April 27, 2023).

[14] National Hospital Daniel Alcides Carrion. Tercera ola de la COVID-19 inició en el Perú. (2022). Available at: https://www.hndac.gob.pe/tercera-ola-de-la-covid-19-inicio-en-el-peru/#:~:text=Ante%20esto%2C%20el%20titular%20de,seguridad%20dentro%20de%20nuestro%20nosocomio (Accessed April 27, 2023).

[15] Essalud. EsSalud: Conoce las variantes causantes del Covid-19 en Perú. Available at: http://noticias.essalud.gob.pe/?inno-noticia=essalud-conoce-las-variantes-causantes-del-covid-19-en-peru#:~:text=Actualmente%2C%20ómicron%20es%20la%20cepa,tercera%20ola%20de%20la%20pandemia (Accessed April 27, 2023).

[16] Ministry of Health, Peru. Coronavirus: variantes de la COVID-19 detectadas en el Perú. (2023). Available at: https://www.gob.pe/12548-coronavirus-variantes-de-la-covid-19-detectadas-en-el-peru (Accessed May 9, 2023).

[17] Seegene. Novaplex™ SARS-CoV-2 variants VII assay. (2023). Available at: https://www.seegene.de/assays/novaplex_sars-cov-2_variants_vii_assay (Accessed May 9, 2023).

[18] Caza M Hogan CA Jassem A Prystajecky N Hadzic A Wilmer A. Evaluation of the clinical and analytical performance of the Seegene allplex™ SARS-CoV-2 variants I assay for the detection of variants of concern (VOC) and variants of interests (VOI). J Clin Virol. (2021) 144:104996. doi: 10.1016/j.jcv.2021.104996, PMID: 34628158 PMC8487322

[19] Vogels CBF Breban MI Ott IM Alpert T Petrone ME Watkins AE. Multiplex qPCR discriminates variants of concern to enhance global surveillance of SARS-CoV-2. PLoS Biol. (2021) 19:e3001236. doi: 10.1371/journal.pbio.3001236, PMID: 33961632 PMC8133773

[20] Padane A Diedhiou CK Gueye K Ndiour S Diagne ND Mboup A. Dynamics of variants of concern (VOC) of SARS-CoV-2 during the different waves of COVID-19 in Senegal. COVID. (2022) 2:691–702. doi: 10.3390/covid2060052

[21] Liotti FM De Maio F Ippoliti C Santarelli G Monzo FR Sali M. Two-period study results from a large Italian hospital laboratory attesting SARS-CoV-2 variant PCR assay evolution. Microbiol Spectr. (2022) 10:e0292222. doi: 10.1128/spectrum.02922-22, PMID: 36409091 PMC9769628

[22] Wei Y Guan J Ning X Li Y Wei L Shen S. Global COVID-19 pandemic waves: limited lessons learned worldwide over the past year. Engineering (Beijing). (2022) 13:91–8. doi: 10.1016/j.eng.2021.07.015, PMID: 34540319 PMC8438800

[23] Justo Arevalo S Uribe Calampa CS Jimenez Silva C Quiñones Aguilar M Bouckaert R Rebello Pinho JR. Phylodynamic of SARS-CoV-2 during the second wave of COVID-19 in Peru. Nat Commun. (2023) 14:3557. doi: 10.1038/s41467-023-39216-8, PMID: 37322028 PMC10272135

[24] Padilla-Rojas C Jimenez-Vasquez V Hurtado V Mestanza O Molina IS Barcena L. Genomic analysis reveals a rapid spread and predominance of lambda (C.37) SARS-COV-2 lineage in Peru despite circulation of variants of concern. J Med Virol. (2021) 93:6845–9. doi: 10.1002/jmv.27261, PMID: 34370324 PMC8427000

[25] Becerra-Flores M Cardozo T. SARSCoV-2 viral spike G614 mutation exhibits higher case fatality rate. Int J Clin Pract. (2020) 74:e13525. doi: 10.1111/ijcp.13525, PMID: 32374903 PMC7267315

[26] Korber B Fischer WM Gnanakaran S Yoon H Theiler J Abfalterer W. Tracking changes in SARS-CoV-2 spike: evidence that D614G increases infectivity of the COVID-19 virus. Cells. (2020) 182:812–827.e19. doi: 10.1016/j.cell.2020.06.043, PMID: 32697968 PMC7332439

[27] Zhu X Mannar D Srivastava SS Berezuk AM Demers JP Saville JW. Cryo-electron microscopy structures of the N501Y SARS-CoV-2 spike protein in complex with ACE2 and 2 potent neutralizing antibodies. PLoS Biol. (2021) 19:e3001237. doi: 10.1371/journal.pbio.3001237, PMID: 33914735 PMC8112707

[28] Tegally H Wilkinson E Giovanetti M Iranzadeh A Fonseca V Giandhari J. Detection of a SARS-CoV-2 variant of concern in South Africa. Nature. (2021) 592:438–43. doi: 10.1038/s41586-021-03402-9 33690265

[29] Tao K Tzou PL Nouhin J Gupta RK de Oliveira T Kosakovsky Pond SL. The biological and clinical significance of emerging SARS-CoV-2 variants. Nat Rev Genet. (2021) 22:757–73. doi: 10.1038/s41576-021-00408-x, PMID: 34535792 PMC8447121

[30] Choi B Choudhary MC Regan J Sparks JA Padera RF Qiu X. Persistence and evolution of SARS-CoV-2 in an immunocompromised host. N Engl J Med. (2020) 383:2291–3. doi: 10.1056/NEJMc2031364, PMID: 33176080 PMC7673303

[31] Gangavarapu K Latiff AA Mullen JL Alkuzweny M Hufbauer E Tsueng G. Outbreak. Info genomic reports: scalable and dynamic surveillance of SARS-CoV-2 variants and mutations. Nat Methods. (2023) 20:512–522. doi: 10.1101/2022.01.27.22269965 36823332 PMC10399614

[32] Hodcroft EB Domman DB Snyder DJ Oguntuyo KY van Diest M Densmore KH. Emergence in late 2020 of multiple lineages of SARS-CoV-2 spike protein variants affecting amino acid position 677. medRxiv. (2021). doi: 10.1101/2021.02.12.21251658, PMID: 33594385 PMC7885944

[33] Emary KRW Golubchik T Aley PK Ariani CV Angus B Bibi S. Efficacy of ChAdOx1 nCoV-19 (AZD1222) vaccine against SARS-CoV-2 variant of concern 202012/01 (B.1.1.7): an exploratory analysis of a randomised controlled trial. Lancet. (2021) 397:1351–62. doi: 10.1016/S0140-6736(21)00628-0, PMID: 33798499 PMC8009612

[34] Heath PT Galiza EP Baxter DN Boffito M Browne D Burns F. Safety and efficacy of NVX-CoV2373 Covid-19 vaccine. N Engl J Med. (2021) 385:1172–83. doi: 10.1056/NEJMoa2107659, PMID: 34192426 PMC8262625

[35] Dagan N Barda N Kepten E Miron O Perchik S Katz MA. BNT162b2 mRNA Covid-19 vaccine in a nationwide mass vaccination setting. N Engl J Med. (2021) 384:1412–23. doi: 10.1056/NEJMoa2101765, PMID: 33626250 PMC7944975

[36] Katz MA Harlev EB Chazan B Chowers M Greenberg D Peretz A. Early effectiveness of BNT162b2 Covid-19 vaccine in preventing SARS-CoV-2 infection in healthcare personnel in six Israeli hospitals (CoVEHPI). Vaccine. (2022) 40:512–20. doi: 10.1016/j.vaccine.2021.11.092, PMID: 34903372 PMC8662353

[37] Nasreen S Chung H He S Brown KA Gubbay JB Buchan SA. Effectiveness of COVID-19 vaccines against symptomatic SARS-CoV-2 infection and severe outcomes with variants of concern in Ontario. Nat Microbiol. (2022) 7:379–85. doi: 10.1038/s41564-021-01053-0, PMID: 35132198

[38] Madhi SA Baillie V Cutland CL Voysey M Koen AL Fairlie L. Efficacy of the ChAdOx1 nCoV-19 Covid-19 vaccine against the B.1.351 variant. N Engl J Med. (2021) 384:1885–98. doi: 10.1056/NEJMoa2102214, PMID: 33725432 PMC7993410

[39] Shinde V Bhikha S Hoosain Z Archary M Bhorat Q Fairlie L. Efficacy of NVX-CoV2373 Covid-19 vaccine against the B.1.351 variant. N Engl J Med. (2021) 384:1899–909. doi: 10.1056/NEJMoa2103055, PMID: 33951374 PMC8091623

[40] Johnson & Johnson. (2021). Johnson & Johnson announces single-shot Janssen COVID-19 vaccine candidate met primary endpoints in interim analysis of its phase 3 ENSEMBLE trial. Available at: https://www.jnj.com/johnson-and-johnson-announces-single-shot-janssen-covid-19-vaccine-candidate-met-primary-endpoints-in-interim-analysis-of-its-phase-3-ensemble-trial (Accessed September 23, 2023).

[41] Abu-Raddad LJ Chemaitelly H Butt AA. National Study Group for Covid-vaccination. Effectiveness of the BNT162b2 Covid-19 vaccine against the B.1.1.7 and B.1.351 variants. N Engl J Med. (2021) 385:187–9. doi: 10.1056/NEJMc2104974 33951357 PMC8117967

[42] Israel Ministry of Health. (2021). Decline in vaccine effectiveness against infection and symptomatic illness. Available at: https://www.gov.il/en/departments/news/05072021-03 (Accessed September 20, 2023.

[43] Tartof SY Slezak JM Heidi F Hong V Ackerson BK Ranasinghe ON. Six-month effectiveness of BNT162b2 mRNA COVID-19 vaccine in a large US integrated health system: a retrospective cohort study. Lancet. (2021) 398:1407–16. doi: 10.1016/S0140-6736(21)02183-8, PMID: 34619098 PMC8489881

[44] Pfizer BioNTech. (2021). Pfizer and BioNTech announce phase 3 trial data showing high efficacy of a booster dose of their COVID-19 vaccine. Available at: https://www.pfizer.com/news/press-release/press-release-detail/pfizer-and-biontech-announce-phase-3-trial-data-showing (Accessed September 18, 2023).

[45] Sheikh A McMenamin J Taylor B Robertson C Public Health Scotland and the EAVE II Collaborators. SARS-CoV-2 Delta VOC in Scotland: demographics, risk of hospital admission, and vaccine effectiveness. Lancet. (2021) 397:2461–2. doi: 10.1016/S0140-6736(21)01358-1, PMID: 34139198 PMC8201647

[46] Lopez Bernal J Andrews N Gower C Gallagher E Simmons R Thelwall S. Effectiveness of Covid-19 vaccines against the B.1.617.2 (Delta) Variant. N Engl J Med. (2021) 385:585–94. doi: 10.1056/NEJMoa2108891, PMID: 34289274 PMC8314739

[47] Pouwels KB Pritchard E Matthews PC Stoesser N Eyre DW Vihta K-D. Effect of Delta variant on viral burden and vaccine effectiveness against new SARS-CoV-2 infections in the UK. Nat Med. (2021) 27:2127–35. doi: 10.1038/s41591-021-01548-7, PMID: 34650248 PMC8674129

[48] Grannis SJ Rowley EA Ong TC Stenehjem E Klein NP DeSilva MB. Interim estimates of COVID-19 vaccine effectiveness against COVID-19-associated emergency department or urgent care clinic encounters and hospitalizations among adults during SARS-CoV-2 B.1.617.2 (Delta) variant predominance – nine states, June-august 2021. MMWR Morb Mortal Wkly Rep. (2021) 70:1291–3. doi: 10.15585/mmwr.mm7037e2, PMID: 34529642 PMC8445373

[49] Andrews N Stowe J Kirsebom F Toffa S Rickeard T Gallagher E. Effectiveness of COVID-19 vaccines against the omicron (B.1.1.529) variant of concern. N Engl J Med. (2022) 386:1532–46. doi: 10.1056/NEJMoa2119451, PMID: 35249272 PMC8908811

[50] Gram MA Emborg H-D Schelde AB Friis NU Nielsen KF Moustsen-Helms IR. Vaccine effectiveness against SARS-CoV-2 infection or COVID-19 hospitalization with the Alpha, Delta, or Omicron SARS-CoV-2 variant: A nationwide Danish cohort study. PLoS Med. (2022) 19:e1003992. doi: 10.1371/journal.pmed.1003992 36048766 PMC9436060

[51] UK Health Security Agency. (2021). Effectiveness of 3 doses of COVID-19 vaccines against symptomatic COVID-19 and hospitalization in adults aged 65 years and older. UK: Department of Public Health.

[52] Public Health England. (2021). SARS-CoV-2 variants of concern and variants under investigation in England: technical briefing: update on hospitalization and vaccine effectiveness for omicron VOC-21NOV-01 (B.1.1.529). UK: Department of Public Health.

[53] Promja S Puenpa J Achakulvisut T Poovorawan Y Lee SY Athamanolap P. Machine learning-assisted real-time polymerase chain reaction and high-resolution melt analysis for SARS-CoV-2 variant identification. Anal Chem. (2023) 95:2102–9. doi: 10.1021/acs.analchem.2c05112, PMID: 36633573

[54] Beguir K Skwark MJ Fu Y Pierrot T Carranza NL Laterre A. Early computational detection of potential high-risk SARS-CoV-2 variants. Comput Biol Med. (2023) 155:106618. doi: 10.1016/j.compbiomed.2023.106618, PMID: 36774893 PMC9892295

[55] Togrul M Arslan H. Detection of SARS-CoV-2 Main Variants of Concerns using Deep Learning 2022. Innovations in intelligent systems and applications conference (ASYU), Antalya, Turkey, 2022, pp. 1–5.

[56] Perez-Romero CA Mendoza-Maldonado L Tonda A Coz E Tabeling P Vanhomwegen J. An innovative AI-based primer design tool for precise and accurate detection of SARS-CoV-2 variants of concern. Sci Rep. (2023) 13:15782. doi: 10.1038/s41598-023-42348-y, PMID: 37737287 PMC10516913

[57] Ferguson N Ghani A Cori A Hogan A Hinsley W Volz E. Report 49—growth, population distribution and immune escape of the omicron in England. MRC Centre for global infectious disease analysis, (2021). London, United Kingdom.

